# Persistent Postoperative Delirium Following Laparoscopic Cholecystectomy

**DOI:** 10.7759/cureus.40523

**Published:** 2023-06-16

**Authors:** Ameya Nair, Sara Arfan, Shaniah S Holder, Kavonne I Bacchus, Timothy J Stear

**Affiliations:** 1 Medicine, Saint James School of Medicine, Arnos Vale, VCT; 2 General Surgery, Windsor University School of Medicine, Chicago, USA; 3 Medicine, American University of Barbados School of Medicine, Bridgetown, BRB; 4 Medicine, Richmond Gabriel University, Chicago, USA; 5 General Surgery, Community First Medical Center, Chicago, USA; 6 General Surgery, Resurrection Medical Center, Chicago, USA

**Keywords:** postoperative period, delirium, cholecystitis, cholecystectomy, altered mental status

## Abstract

Gallstones are the primary cause of symptomatic gallbladder disease and lead to a significant portion of hospitalizations related to gastrointestinal diseases. The gold standard treatment for gallbladder disease continues to be cholecystectomy, which is commonly done laparoscopically, and improves patients’ quality of life. With any surgical intervention there are inherent risks, and in the setting of severe illness, the risk of potential complications increases immensely. Postoperative altered mental status, namely, delirium, may occur in the elderly and a high index of suspicion is required to recognize the clinical signs for swift diagnosis and management. This case involves a 61-year-old male who underwent laparoscopic cholecystectomy and developed persistent delirium during the hospital course. This report aims to explore the multiple risk factors that lead to postoperative delirium and review the diagnostic and therapeutic strategies utilized in managing this patient.

## Introduction

Approximately 38 million people in the United States (US) have gallstones; however, most cases are asymptomatic and do not require treatment [1]. Some common symptomatic complications of gallstones that require management include cholecystitis, cholangitis, choledocholithiasis, and pancreatitis [1]. These diseases continue to be some of the leading causes of hospitalizations related to gastrointestinal issues in developed countries [2].

Cholecystitis is characterized by obstruction of the cystic duct, most commonly by a stone, which leads to inflammation of the gallbladder [3]. It is seen in 1%-3% of persons with gallstones, and patients usually present with right upper quadrant (RUQ) pain that may radiate to the scapula, vomiting, and fever [1,3]. A positive Murphy's sign is indicative of this condition and can be elicited during physical exam or ultrasound (U/S), which is the initial diagnostic test of choice [1]. Laparoscopic cholecystectomy is the gold standard mode of management for symptomatic gallbladder diseases with about 750,000 operations performed annually in the US [2]. This procedure is minimally invasive and is associated with a lower procedure risk, decreased length of hospital stay, and improved healing outcome. Although this procedure is normally safe, the severity of illness upon presentation may affect the surgery itself and lead to many postoperative complications such as altered mental status.

Altered mental status describes a change in cognition, mood, or behavior, and differentiates into stupor, coma, delirium, or encephalopathy [4]. The risk of postoperative delirium (POD) is higher in the elderly, affecting up to 50% of persons, and can be precipitated by increased surgery duration, severe infection, electrolyte imbalance, and medication use such as narcotics or anesthetics [5]. Delirium occurs within hours to days of the inciting trigger, resulting in disorientation with respect to person, place, or time, forgetfulness, aggression, hallucinations or delusions, distractibility, and incoherent or nonsensical speech [5]. The recovery time of delirium is variable and depends on how quickly the precipitating factor is treated. In some cases, delirium may persist despite appropriate management of the possible underlying causes.

This case report presents a patient with U/S-confirmed cholecystitis who underwent a laparoscopic cholecystectomy. Two days after the procedure, the patient became confused, disoriented, and agitated and was subsequently diagnosed with delirium. Despite appropriate medical optimization, the patient’s condition continued to deteriorate with prolonged delirium that led to institutionalization.

## Case presentation

A 61-year-old Hispanic male presented to the emergency department (ED) with abdominal pain and intractable emesis for one week. His past medical history was significant for hyperlipidemia, hypertension, and diabetes mellitus. The patient complained of fatigue, chills, dizziness, and intermittent epigastric pain accompanied by nausea and vomiting. He denied a history of illicit drug or alcohol use. En route to the hospital, the patient became unresponsive and hypotensive with a systolic blood pressure (SBP) in the 80s that responded to intravenous fluids. On admission, the patient was diaphoretic, ill-appearing, hypotensive, tachycardic and confused. Vital signs were unstable with a temperature of 100.1 °F, pulse of 92 bpm, respiratory rate (RR) of 31 bpm, blood pressure of 105/66 mmHg and oxygen saturation of 95% on room air.

On physical examination, the patient was alert and oriented to person, place, and time. His abdomen was diffusely tender to palpation in all quadrants without guarding or rigidity. There were no external signs of injury and he denied any traumatic events, headaches, or neck stiffness. The preliminary investigation revealed elevated venous lactate, total bilirubin, and alkaline phosphatase levels. Laboratory values on admission are demonstrated in Table 1.

**Table 1:** Patient's laboratory values on presentation to the emergency department AST: aspartate aminotransferase; ALT: alanine aminotransferase

Laboratory test	Patient's laboratory values	Reference range	Interpretation
Lactate	6.6 mmol/L	0.5-2.0 mmol/L	Elevated
White blood cell count	8.5 x 10^9^/L	4.5-11.0 x 10^9^/L	Within normal limits
Glucose	159 mmol/L	70-99 mmol/L	Elevated
Procalcitonin	5.30 ng/mL	0.20-0.49 ng/mL	Elevated
Troponin	<0.03 ng/mL	0.0-0.04 ng/mL	Within normal limits
Protein	5.4 g/dL	6-8 g/dL	Within normal limits
Albumin	3.0 g/L	3.4-5.4 g/L	Decreased
Total bilirubin	3.0 mg/dL	0.0-1.0 mg/dL	Elevated
Alkaline phosphatase	191 IU/L	40-129 IU/L	Elevated
AST	42 IU/L	13-39 IU/L	Elevated
ALT	55 IU/L	7.0-52 IU/L	Elevated
Sodium	130 mEq/L	135-145 mEq/L	Decreased
Potassium	4.2 mEq/L	3.5-5.0 mEq/L	Within normal limits
Chloride	91 mEq/L	96-106 mEq/L	Decreased
Magnesium	4.5 mg/dL	1.7-2.2 mg/dL	Elevated

**Figure 1:**
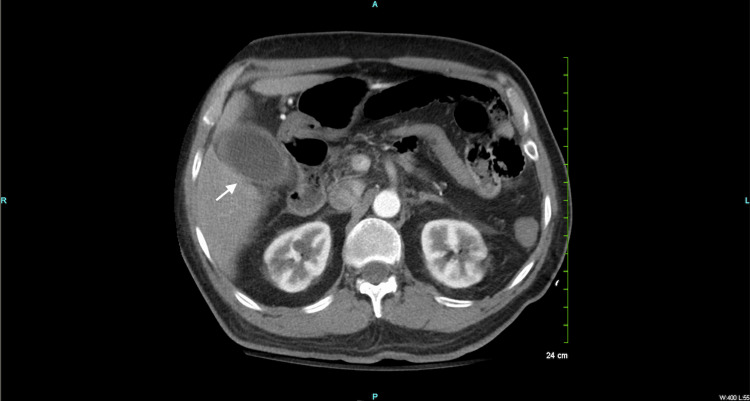
Axial contrast-enhanced computed tomography of the abdomen and pelvis demonstrating gallbladder dilation with wall thickening and fat stranding (white arrow)

Given the positive physical findings, transaminitis, and elevated total bilirubin, computed tomography (CT) of the abdomen and pelvis with intravenous (IV) contrast was conducted. Results revealed a dilated gallbladder with wall thickening and fat stranding, consistent with acute cholecystitis (Figure 1).

**Figure 2:**
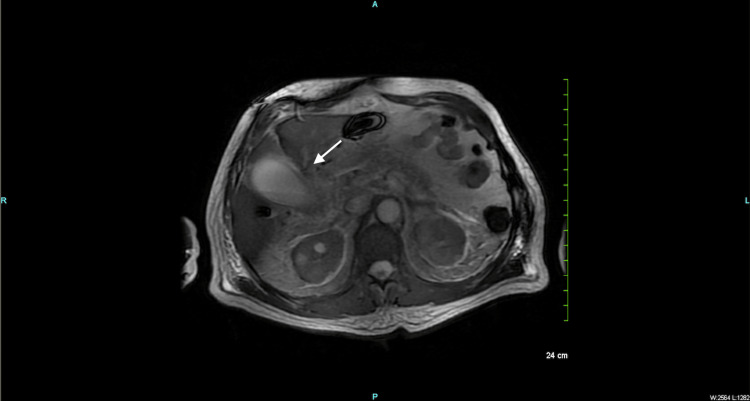
Axial view of magnetic resonance cholangiopancreatography shows biliary sludge (white arrow)

The patient was diagnosed with sepsis and fever of unspecified origin and was placed on a regimen of piperacillin-tazobactam. Magnetic resonance cholangiopancreatography (MRCP) was ordered for suspected choledocholithiasis. Imaging revealed a borderline dilated common bile duct (CBD), pericholecystic fluid and biliary sludge, but there was no evidence of choledocholithiasis (Figure 2).

Due to these diagnostic findings, the patient underwent laparoscopic cholecystectomy with an intraoperative cholangiogram. The surgery was conducted under general anesthesia and the duration was less than one hour. During the operation, the gallbladder was distended and severely inflamed, which was subsequently decompressed with a laparoscopic decompression needle. Purulent bile was aspirated and sent for culture. Due to the severe inflammatory changes, extensive careful dissection was required to safely identify the cystic artery and duct. Intraoperative cholangiography was performed as planned for elevated liver enzymes and dilated CBD with normal cholangiogram findings. The surgery was completed successfully without any intraoperative complications. The intraoperative blood loss was estimated to be 20 mL.

**Figure 3:**
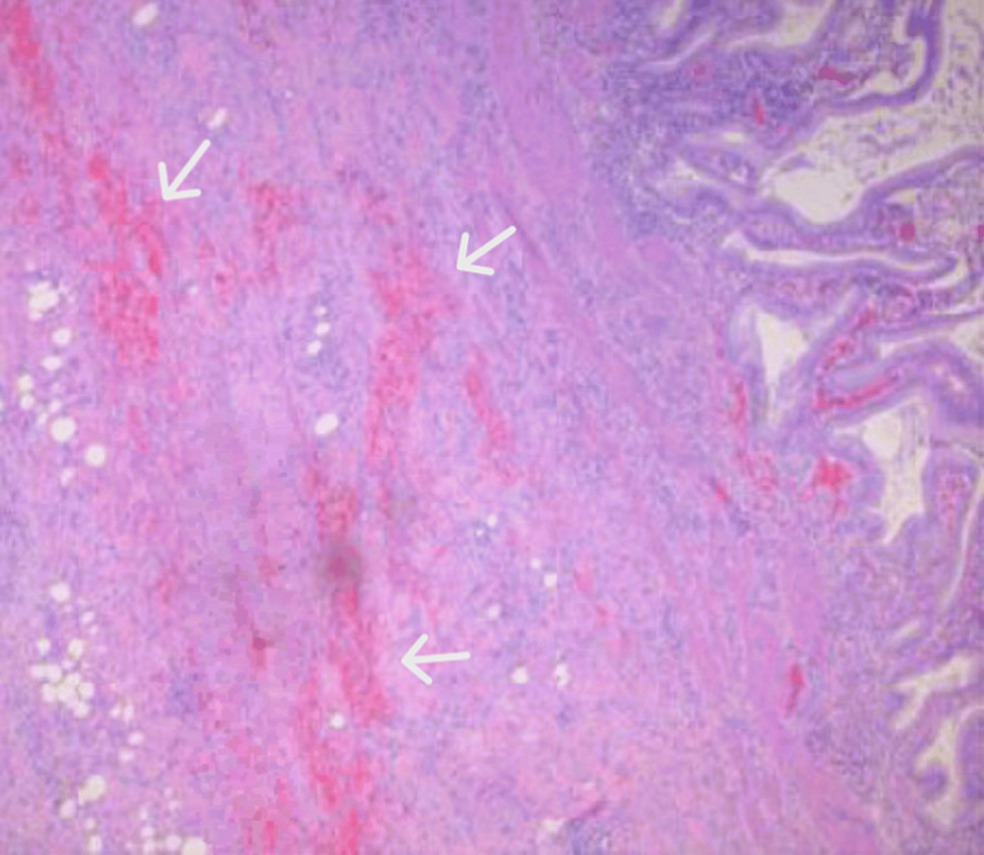
Hematoxylin and eosin (H&E) stain of the gallbladder, demonstrating focal hemorrhagic and reactive changes (white arrows)

The patient was neurologically alert and responsive during the immediate postoperative period. He denied abdominal pain, chills, shortness of breath, nausea, or vomiting postoperatively. Pathological evaluation of the gallbladder specimen revealed a thickened gallbladder wall measuring 0.5 to 0.8 cm, hemorrhagic bile, and focal hemorrhagic and reactive cellular changes consistent with moderate to severe acute on chronic cholecystitis (Figure 3).

On postoperative day 1, the patient was seen and examined by the medical and surgical team. The patient had no overnight events and only endorsed mild incisional pain on physical examination. He denied any nausea or vomiting.

On postoperative day 2, the patient became severely agitated, confused, disoriented, and restless. He removed his Foley catheter resulting in significant bleeding, and attempted to pull out his IV line. The patient stated that he was waiting at a train station and believed it was the year 2007, indicating he was not oriented to person, place, or time. A repeat electrocardiogram (ECG) was significant for paroxysmal atrial fibrillation (AFib) with rapid ventricular response (RVR) in the 180s. The patient’s blood pressure skyrocketed to 171/113 mmHg and his pulse fluctuated between 120 and 150 bpm.

**Figure 4:**
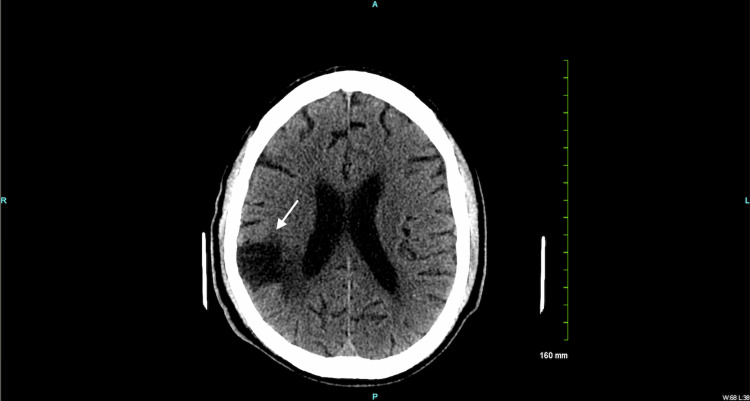
Non-contrast head CT demonstrating focal encephalomalacia (white arrow)

On postoperative day 3, the patient had multiple hypertensive episodes despite appropriate management. He also became combative and was placed in soft restraints. A one-time dose of 5.0 mg intramuscular (IM) haloperidol was administered with an additional dosage of 3.0 mg when needed. On assessment, his speech was limited and he was only oriented to person but not time or place, which was below his baseline. The head CT without contrast revealed focal encephalomalacia and volume loss within the right parietal and left occipital lobe (Figure 4).

**Figure 5:**
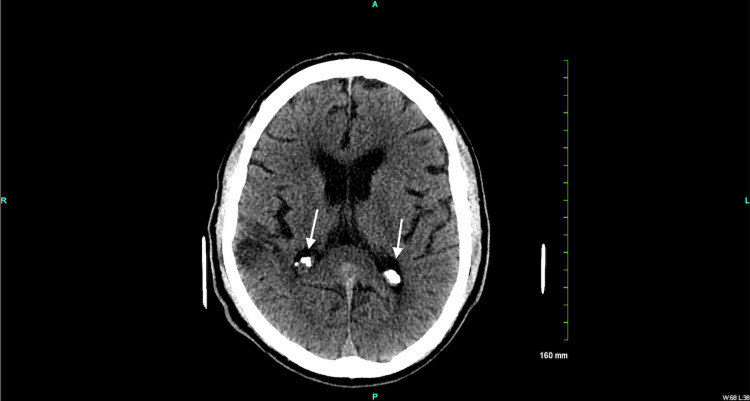
Non-contrast head CT demonstrating intracranial atherosclerotic calcifications (white arrows)

There was evidence of old bilateral infarcts and intracranial atherosclerotic calcification (Figure 5).

There was no evidence of an acute intracranial process, intracranial hemorrhage, mass effect, or midline shift. Despite appropriate conservative management, the patient continued to exhibit these features. Therefore, on postoperative day 5, an electroencephalogram (EEG) was conducted. Findings revealed diffuse disorganization of the cerebral process that prompted the use of a head CT scan.

On postoperative day 9, the patient had continued symptoms of delirium. He was discharged to a skilled nursing facility for continued care as his medical status no longer warranted acute hospitalization. At the time of discharge, the patient had no electrolyte disturbances, fever or signs of sepsis. Acute intracranial processes were ruled out by CT. Instructions were provided to follow up with his primary care physician in two weeks.

## Discussion

Delirium is defined as an altered mental status that results in confusion and lack of awareness of either person, place, or time [6]. Delirium can be classified as an acute cognitive impairment but is separate from dementia, which is classified by a long-term mental functional decline and confusion. Delirium can be classified into three subtypes based on psychomotor activity and levels of arousal: hyperactive, hypoactive, and mixed. The prevalence of delirium in the community setting is only 1%-2%; however, the risk of development increases with age and existing comorbidities that may lead to hospitalization [7]. In hospitalized patients over the age of 65, delirium is the most common complication in the United States. Postoperative delirium is a type of delirium that develops after a patient undergoes surgery, and poses a great risk for elderly patients.

A variety of underlying age-dependent structural changes in the brain can cause postoperative delirium, which can be precipitated by advanced age [8]. Brain shrinkage is a well-documented age-related process that involves many cellular and subcellular changes. Loss of dendritic spines and shrinkage of large pyramidal neurons are associated with a loss of up to 45% of myelinated axons in persons by the age of 80 [9]. These changes reduce the brain’s capacity to fulfill its physiological role, and as a result, there is reduced resilience in the presence of neurological insult. Some research has shown that abnormalities in thalamic inputs to the amygdala, hypothalamus, and periaqueductal gray matter play a role in the development of persistent POD [8]. Thalamus-mediated impairment may present with slow wave activity on EEG and global reduction in cerebral blood flow [10]. Systemic inflammation due to infection, trauma, or surgery can also contribute to the pathophysiology of POD development [11]. The inflammatory activation of primed microglia leads to increased levels of cytokines in the brain and results in the disruption of synaptic communication [10]. Postoperative delirium can develop in patients due to neurological insults from various and often compounding etiological factors.

In postoperative patients, delirium occurs in 15%-53% of elderly patients and 70%-87% of intensive care unit (ICU) patients [12]. In a study conducted by Robinson et al., 144 patients over the age of 50 who underwent major surgery were observed [13]. This study reported postoperative delirium in 44% of study participants and concluded that preexisting cognitive dysfunction was the strongest predictor of POD development. Some researchers postulate that the wide range of POD incidences in the literature may be due to the various baseline cognitive defects and neuropsychiatric disorders in patients [14]. The patient in our case had a mild cognitive defect at baseline that may have increased his risk of developing POD.

Other risk factors include male sex, decreased fluid intake, and multidrug therapy, particularly psychoactive medications [15]. Within the acute care surgery population, the use of a Foley catheter, ICU admission, and frailty were found to be independent risk factors for persistent postoperative delirium [14]. Surgeries involving the biliary tract, gastrointestinal surgery, and appendectomy increased the risk of POD. In this case, the delirium was likely due to severe cholecystitis with sepsis, prolonged surgery duration, and anesthesia use. The patient also had a history of atrial fibrillation managed with Eliquis at home. The presence of AFib and old cerebral infarcts found on head CT may also have contributed to his persistent POD. This could suggest that existing cerebrovascular insults could reduce the brain's resilience to recover from delirium associated with sepsis and anesthesia use.

The clinical diagnosis of delirium can be made using the Confusion Assessment Method (CAM) that requires an acute change in mental status, inattention, and the presence of either disorganized thinking or altered level of consciousness [16]. EEG may be used to understand the nature of the neuronal involvement. In some delirious patients, the EEG can be used to indicate whether the patient is suffering from focal or global impairment [17]. Patients with delirium may have an irregular background rhythm, widespread theta or delta slow wave activity, slowed posterior dominant rhythm, and decreased sensitivity to eye opening and closing on their EEGs. In our case, the patient’s EEG showed severe slowing and diffuse disorganization of the cerebral process that was consistent with his clinical diagnosis.

Most cases of POD are acute and resolve within one day post-operation [18]. In this patient, the delirium did not resolve during the hospital course. This adverse outcome of persistent delirium may lead to long-term dementia and a worsened prognosis in elderly patients [18]. Elderly patients may meet the criteria for the diagnosis of delirium up to one year after the operation and a preoperative level of consciousness may not be restored [18,19]. A study by Rockwood et al. found that the risk of developing dementia after a delirium episode was three times higher than in the general population [20]. Among hospitalized patients with delirium, the mortality rates can significantly increase [19]. In our case, the patient will not continue to be followed up by our team after his discharge to a skilled nursing facility, and therefore, long-term implications of his POD cannot be assessed.

The treatment of POD often involves addressing the inciting factors and stabilizing the patient. The non-pharmacological approach includes reorientation and behavioral intervention [11]. Caregivers and clinicians should use clear instructions and make frequent eye contact with patients, avoid the use of restraints as they increase agitation, and mediate any sensory impairments. Pharmacological strategies are still being debated; however, some clinicians encourage the use of typical or atypical antipsychotics, benzodiazepines, and cholinesterase inhibitors for acute and prophylactic therapy [11]. For patients with persistent delirium refractory to medical stabilization, supportive care may become the terminal option.

The patient reported here had several risk factors for developing persistent postoperative delirium, including his gender, severe illness, anesthesia use, existing AFib and a history of old cerebral infarcts. One limitation of this case report is that the patient's baseline alertness outside of the hospital was not well documented or established prior to ED evaluation. We relied on the testimony of family members to understand his normal cognitive function at home prior to hospitalization.

## Conclusions

Postoperative delirium is a severe cognitive impairment that affects the elderly and is associated with multiple risk factors. Persistent POD that does not resolve during the hospital course increases the risk of significant adverse effects such as dementia. It can also lead to institutionalization and increased mortality rates in elderly patients. In addition, POD poses a significant financial burden on the healthcare system.

Further research is required on the pathophysiology of persistent POD to understand why some cases do not resolve during the hospital course and may extend into dementia. Further research may also help us understand how old cerebral infarcts can reduce resilience to postoperative delirium. Physicians should also be aware of the symptoms of POD and use diagnostic tools such as the Confusion Assessment Method in order to detect delirium. Prompt detection is important as it allows for appropriate diagnostic tests to be ordered and aids in identifying the precipitating factors. Knowledge of the preventative and therapeutic strategies is paramount to shortening the clinical course of delirium to improve the prognosis and the patient’s quality of life.
